# Supplementary material for: Complexome profiling of the Chlamydomonas psb28 mutant reveals TEF5 as an early PSII assembly factor
Source: Plant Cell. 2025 Mar 17;37(6):koaf055. doi: 10.1093/plcell/koaf055 (PMC12164590; doi:10.1093/plcell/koaf055)
Supplement: koaf055_Supplementary_Data [file koaf055_supplementary_data.zip › psb28_interactive.zip!/plots/intensity/qualityPlots/peptides/CPLD20_Cre01.g000900.t1.2.html]

### Cre01.g000900.t1.2

Trivial name: CPLD20  
SeqCoverage [median]: 20.70  
Mass: 50.84 kDa  
  
 

| Sequence | MissedCleavage | Mass [Da] | wtIntensity | muIntensity | Score |
| --- | --- | --- | --- | --- | --- |
| AEFLPTPEETR | 0 | 1288.63 | 2335.46 | 11054.57 | 89.05 |
| GLSPSLVR | 0 | 827.49 | 0.00 | 4982.96 | 97.07 |
| IADEAQFR | 0 | 948.47 | 1908.72 | 12363.30 | 111.82 |
| IADEAQFRFDR | 1 | 1366.66 | 0.00 | 5805.93 | 66.60 |
| LAAGAVESTGGALGR | 0 | 1328.70 | 4010.95 | 18558.70 | 238.92 |
| TLPGDHLRPLHQAFVDLPPDLAR | 1 | 2577.38 | 0.00 | 3894.32 | 44.63 |
| TTVESISETVK | 0 | 1192.62 | 2881.20 | 15853.76 | 150.35 |
| TYYGVSR | 0 | 844.41 | 0.00 | 2738.26 | 89.14 |
| VLGPIINQIAASPVR | 0 | 1546.92 | 2809.22 | 11466.13 | 145.31 |
|  |  |  | 13945.6 | 86717.9 |  |
